# Stromal cells help ATL cells to escape from apoptosis induced by histone deacetylase inhibitors

**DOI:** 10.1186/1742-4690-8-S1-A167

**Published:** 2011-06-06

**Authors:** Yukiko Miyatake, Utano Tomaru, André L A Oliveira, Noreen Sheehy, Takashi Yoshiki, Masanori Kasahara, William W Hall

**Affiliations:** 1 Department of Pathology, Hokkaido University Graduate School of Medicine, Sapporo, 060-8638, Japan; 2 Centre for Research in Infectious Diseases, School of Medicine and Medical Science, University College Dublin, Dublin, 4, Ireland

## 

HTLV-1 expression is generally extremely low in freshly isolated ATL cells, suggesting that gene silencing has occurred. The silencing of viral genes in ATL cells could be due to not only host epigenetic mechanism, but also consequences of natural selection of ATL cells to evade the host immune response. Recently, it has been reported that stromal cells could contribute to the regulation of HTLV-1 expression. In this study, we investigated whether stromal cells could be involved in reactivation of viral gene expression in HTLV-1 infected cells using co-culture systems. We used CR cells, a viral gene silenced ATL cell line, and human HEK-293T epithelial cells as stromal cells. To reactivate viral gene expression in CR cells, the histone deacetylase inhibitor, Trichostatin A (TSA) was employed. CR cells co-cultured with HEK-293T cells dramatically escaped TSA-induced apoptosis, while CR cells cultured alone underwent apoptosis by TSA in a dose-dependent manner. Moreover, the transcriptional activity of NF-κB in CR cells co-cultured with HEK-293T cells was decreased by TSA, whereas the activity in CR cells cultured alone was increased. These results suggest that stromal cells might allow HTLV-1-infected cells to escape from apoptosis induced by activation of HTLV-1 expression. This study provides an insight into the potential role of stromal cells in the maintenance of latency in HTLV-1 infected cells.

